# The Royal Society of Medicine

**Published:** 1915-09-25

**Authors:** 


### THE ROYAL SOCIETY OF MEDICINE.

The Royal Society of Medicine may now be confidently regarded as having passed successfully through the dangers which proverbially attend a new enterprise, and especially a new enterprise in a profession of distinctly conservative tendencies and established habits. The Society came into existence with difficulty, and its advent was not unaccompanied by prophecies of evil and disaster. Not unnaturally it excited jealousies, as its appearance was in some degree a challenge to organisations actually in operation and proud of their successes and traditions. Time has justified the vision and enterprise of the founders, and to-day, if the critics have not wholly disappeared, they have fallen to a very limited few. Even on the financial side, where from the first there were real anxieties, the position is not unsatisfactory. On the last year's working there is a balance of over £800, while the capital account shows a debt of £26,000, against which there are, of course, substantial assets.

In reference to the financial position, we are glad to note the absence of further appeal to the public. The times are, of course, not propitious for such appeals, and personally we were never able to sympathise with the Society's suggestion that there was a case for outside help. The organisation is a domestic one, and its claim is on the profession. No doubt it may be argued that by helping to maintain and increase the efficiency of the medical profession it contributes to the public welfare. But the same may be said of any respectable professional organisation whether relating to medicine or to other ambitions, and thus in our judgment some of the earlier efforts to raise money were decidedly wanting both in substance and in dignity. The suggested Insurance Company Association was a particularly unfortunate proposal, and happily it was rejected by the good sense of the Fellows, who considerately built a bridge over which the originators of the scheme were able to execute a graceful movement of retreat. It is pleasing to know that all these extraneous and artificial aids have proved to be unnecessary, and that the Society can pursue its career without obligations outside its own organisation.

What the Society has done and is doing cannot be stated in a paragraph. It has given the profession a home in the Metropolis both dignified in appearance and convenient in arrangement. The resources of its library and reading-room are excellent, if not quite complete. For meetings, both sectional and general, it provides a very satisfactory equipment, and it is able to encourage a certain amount of research work. Much more important is the influence it exercises in its indirect encouragement to careful observation and record among its Fellows, and the centralising force it exercises over its various sections. Prior to the existence of the Royal Society individual organisations representing the various activities of the profession were isolated and detached from one another, and there was the danger which is inevitable in such isolation. Now this danger is at least in part corrected, and individual movements are kept more or less in touch with the main stream. This is an important achievement and deserves warm recognition. To say that the Society has now nothing to do but to rest on its oars would be to pay it a poor compliment. On the contrary, we hope both for improvements and developments, and we feel sure this is the conviction of those who have done most for the Society and are most concerned for its welfare. It is scant justice to say that the Fellows owe a debt of gratitude to Mr. J. Y. W. MacAlister, the Secretary, and we are sure that this is widely acknowledged.

